# Variations of a group coaching intervention to support early-career biomedical researchers in Grant proposal development: a pragmatic, four-arm, group-randomized trial

**DOI:** 10.1186/s12909-021-03093-w

**Published:** 2022-01-10

**Authors:** Anne Marie Weber-Main, Jeffrey Engler, Richard McGee, Marlene J. Egger, Harlan P. Jones, Christine V. Wood, Kristin Boman, Jiqiang Wu, Andrew K. Langi, Kolawole S. Okuyemi

**Affiliations:** 1 Department of Medicine, University of Minnesota Medical School, Minneapolis, MN 55455 USA; 2 San Diego, USA; 3 Faculty Affairs, Northwestern University Feinberg School of Medicine, Chicago, IL 60611 USA; 4 Department of Family and Preventive Medicine, Division of Public Health, University of Utah School of Medicine, Salt Lake City, UT 84108 USA; 5 Center for Diversity and International Programs, University of North Texas Health Science Center, Fort Worth, TX 76107 USA; 6 Department of Medical Social Sciences, Northwestern University Feinberg School of Medicine, Chicago, IL 60611 USA; 7 Department of Family and Preventive Medicine, University of Utah, Salt Lake City, UT 84108 USA; 8 Department of Family and Preventive Medicine, Division of Public Health, University of Utah, Salt Lake City, 84108 USA

**Keywords:** Biomedical research, Research proposal, National Institutes of Health, Grants, Writing, Mentoring, Coaching, Underrepresented minorities, Intervention, Pragmatic randomized trial

## Abstract

**Background:**

Funded grant proposals provide biomedical researchers with the resources needed to build their research programs, support trainees, and advance public health. Studies using National Institutes of Health (NIH) data have found that investigators from underrepresented groups in the biomedical workforce are awarded NIH research grants at disproportionately lower rates. Grant writing training initiatives are available, but there is a dearth of rigorous research to determine the effectiveness of such interventions and to discern their essential features.

**Methods:**

This 2 × 2, unblinded, group-randomized study compares the effectiveness of variations of an NIH-focused, grant writing, group coaching intervention for biomedical postdoctoral fellows and early-career faculty. The key study outcomes are proposal submission rates and funding rates. Participants, drawn from across the United States, are enrolled as dyads with a self-selected scientific advisor in their content area, then placed into coaching groups led by senior NIH-funded investigators who are trained in the intervention’s coaching practices. Target enrollment is 72 coaching groups of 4–5 dyads each. Groups are randomized to one of four intervention arms that differ on two factors: [1] duration of coaching support (regular dose = 5 months of group coaching, versus extended dose = regular dose plus an additional 18 months of one-on-one coaching); and [2] mode of engaging scientific advisors with the regular dose group coaching process (unstructured versus structured engagement). Intervention variations were informed by programs previously offered by the NIH National Research Mentoring Network. Participant data are collected via written surveys (baseline and 6, 12, 18, and 24 months after start of the regular dose) and semi-structured interviews (end of regular dose and 24 months). Quantitative analyses will be intention-to-treat, using a 2-sided test of equality of the effects of each factor. An inductive, constant comparison analysis of interview transcripts will be used to identify contextual factors -- associated with individual participants, their engagement with the coaching intervention, and their institutional setting – that influence intervention effectiveness.

**Discussion:**

Results of this study will provide an empirical basis for a readily translatable coaching approach to supporting the essential grant writing activities of faculty, fellows, and other research trainees, including those from underrepresented groups.

**Supplementary Information:**

The online version contains supplementary material available at 10.1186/s12909-021-03093-w.

## Background

The ability to acquire external research funding is an essential skill that biomedical research trainees must develop to make the career transition into independent investigators. This transition is particularly critical for research-intensive faculty in the biomedical sciences, for whom the acquisition of major grant funding from the National Institutes of Health (NIH) or similar federal sources is often a requirement for promotion and tenure. Women and members of some racial/ethnic groups continue to be underrepresented in biomedical faculty positions, particularly at advanced ranks [1]. Among the likely contributors to this inequity are the well-documented disparities in the rates of NIH research proposals that are submitted by, and grants that are awarded to, members of these under-represented groups [2–4].

In 2014, the NIH established the Diversity Program Consortium [5] to enhance the participation and persistence of individuals from underrepresented backgrounds in biomedical research careers. A core component of the Consortium is the National Research Mentoring Network (NRMN) [6]. From 2014 through 2019 (NRMN phase 1), the Professional Development Core of NRMN implemented four different grant writing coaching models for diverse cohorts of early-career investigators across the United States [7, 8]. The models were similar, in that all had an initial in-person training session, followed by virtual coaching meetings (group and/or individual) for several months. Coaching was provided by accomplished investigators with high levels of expertise in NIH proposal writing and reviewing. However, the models differed in several of their core design features, such as trainee eligibility (readiness to write, extent of mentorship in their research area), program duration (4–12 months), type of feedback provided (written, oral, or both), proposal sections covered, and use of mock review. These differences enabled us to descriptively compare preliminary outcomes and process data across the model variations.

A total of 545 individuals (67% female, 61% under-represented racial/ethnic minority, URM) from 187 different institutions participated in at least one of the phase 1 NRMN grant writing coaching models [8]. Irrespective of model, the majority of participants reported meaningful learning gains in their knowledge, skills, and grant writing self-efficacy [9]. Proposal submission and funding outcomes were also positive. For all models combined, nearly 60% of participants submitted at least one grant application within 18 months of program completion, of which 41% were funded. However, we also observed substantial variation in proposal outcomes by model; for example, funding rates among submitters ranged from 24 to 59% across programs [8]. Our examination of phase 1 data and the common/unique features across the models yielded several important insights. First, a ‘single dose’ may be insufficient to achieve funding for all but a subset of participants; many may need sustained coaching beyond the initial intervention period, especially to support proposal resubmissions. Second, input from relevant scientific experts is essential, not just writing coaching. Third, coaching groups appear most effective when they have the following features: participants work through the entire proposal, rather than just a ‘light touch’ focus on early sections of a proposal; groups are composed of peers and coaches aligned by discipline and/or methodology; common writing goals and deadlines are articulated to spur progress; and the coaching experience ends with independent mock review of developed drafts.

We applied these insights to design what we postulate is a more optimized group coaching intervention, which we are currently testing in a pragmatic randomized trial during phase 2 of NRMN (2019–2024). In this intervention, all participants in a coaching group are engaged in similar types of research and/or methodology. The group follows a well-defined 5-month coaching schedule with specified writing assignments. There is an explicit goal of producing a (nearly) complete draft of the core scientific content by the end of the intervention, one that is developed enough to undergo pre-submission mock review. Lastly, each participant identifies what we are calling a “scientific advisor” -- a past or current mentor, a more senior colleague, or a near-peer with expertise in their research content area -- who agrees to provide ongoing scientific feedback on the developing grant proposal as a complement to the intensive group coaching process.

Despite having strong preliminary data to support the value of group coaching in general and to inform our new intervention design, we lacked information about some key variables needed to support broad dissemination of our approach. First, what duration of the coaching intervention is most effective? Second, is there a benefit to a participant’s scientific advisor being directly involved with the group coaching intervention itself? Third, what contextual factors (associated with individual participants, their engagement with the coaching intervention, and their institutional setting) might influence the intervention’s effectiveness? To address these salient research questions, we designed a 2 × 2, 4-arm randomized trial. Aim 1 is to determine the effectiveness of our enhanced group coaching intervention on proposal submission and funding rates when varying the coaching dose and the mode of engaging participants’ scientific advisors with the coaching group. Aim 2 is to identify individual (person), coaching group, and institutional factors that predict proposal submission, resubmission, and funding.

Our study’s potential impact is expected to be high, given the limited systematic studies of interventions specifically aimed at promoting biomedical grant proposal submission and funding acquisition. Although grant writing training for biomedical researchers is not new (professional development opportunities are provided by institutions, funding agencies, scientific societies, and even the private sector), most are limited in their duration and scope, ranging from brief workshops to week-long programs with different training priorities [10, 11]. In some settings, grant writing training is addressed in graduate-level coursework [12–14] or embedded into more comprehensive, multi-year research training programs [15–19]. The wide variability in training approaches has made it difficult to identify and disseminate evidence-based practices. Further, only a few programs with an explicit focus on the proposal development process have reported their intervention procedures and effectiveness data, with or without a comparison group [20–24]. Our study will identify the importance of key features of a pragmatic and readily scalable coaching intervention, while yielding a deeper understanding of why, how, for whom, and in what contexts the intervention is effective.

## Methods/design

### Study setting

The primary study site is the University of Utah School of Medicine. Participants are drawn from a national (United States) population of early-career researchers in the biomedical sciences, with particular attention paid to recruiting individuals from groups underrepresented in the biomedical workforce [25].

### Trial design

This is a 2 × 2, unblinded, group-randomized intervention trial, designed to test the influence of two factors on the grant proposal submission rates and funding rates of aspiring independent biomedical researchers. Factor 1 is the duration of grant writing coaching support that participants receive: regular dose (5 months of group-based coaching) versus extended dose (regular dose plus an additional 18 months of one-on-one coaching). Factor 2 is the mode of engaging participants’ self-selected scientific advisors with the 5-month group coaching process (unstructured versus structured engagement). We hypothesize that significantly higher rates of grant funding will be realized for participants who experience an extended dose of coaching and/or structured engagement of their scientific advisors with the group coaching process. We are applying a mixed method approach that leverages qualitative interviews (with participants and coaches) with quantitative surveys (of participants, coaches, and scientific advisors) and objective outcome data (proposals submitted, funded/unfunded) to elucidate the mechanisms behind any observed differences between and within study arms.

**Fig. 1:**
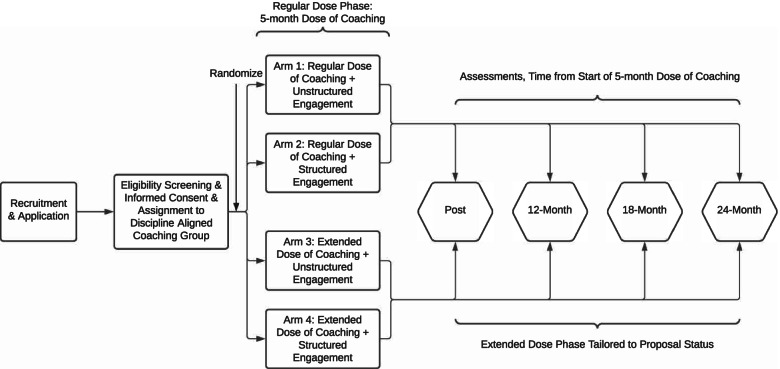
Overview of study design for the University of Utah Grant Writing Coaching Groups Study

Figure 1 illustrates key features of our study. Participants and their scientific advisors are enrolled into the study as dyads and placed into coaching groups of 4 to 5 dyads each. Our enrollment target is 72 coaching groups, distributed over a maximum of 6 cohorts (see *Adequacy of Sample Size* section). Within each cohort, whole coaching groups are randomized to one of four study arms: 1) regular dose + unstructured engagement of scientific advisors; 2) regular dose + structured engagement of scientific advisors; 3) extended dose + unstructured engagement of scientific advisors; or 4) extended dose + structured engagement of scientific advisors. All arms engage participants in “active treatment.” The trial does not include a “no treatment” control arm, because the value of sustained grant writing coaching as a core intervention was demonstrated in our evaluation of the previous NRMN phase 1 models [8]. This study was designed to compare variations of a single, well-defined, group coaching model to identify specific factors that influence its effectiveness. Participants are followed for 24 months to assess their proposal writing activity, grant application outcomes, and related outcomes as described below.

To test the influence of the two intervention factors separately, we designed the study so that all participants first receive the regular coaching dose. This intervention consists of 5 months of group coaching, during which participants develop drafts of different sections of a grant application, then meet as a small group every other week (8 ~ two-hour sessions) to discuss and receive feedback on their work-in-progress from a skilled coach and other group members. During the regular dose, the only difference between study arms is whether participants’ scientific advisors directly engage with the group coaching intervention (structured) or have no direct interaction with the coaching process (unstructured). Group randomization assignment to the structured or unstructured condition is revealed at the start of the regular dose coaching period.

After the regular dose ends and post-intervention data collection is complete, the coaching groups’ randomization to regular dose or extended dose is revealed. If a group is randomized to the regular dose, all coaching support ends. If a group is randomized to the extended dose, the coach continues to support each participant in their group on an individual basis over the next 18 months (a maximum of 10 h per participant). This extended dose of coaching allows participants to receive individualized help with finishing their proposal if needed and/or with crafting a revision of their proposal for resubmission. Additional protocol details for the study arms are provided below.

### Coaching protocols for regular dose and extended dose arms

#### Regular dose coaching protocol

##### Kickoff session

For each study cohort, the regular dose coaching period begins with a 2-day kickoff session for participants and coaches (see Additional File 1 for kickoff agenda). Scientific advisors do not attend. Separate sessions are held for coaching groups in the structured engagement and unstructured engagement arms. Originally, kickoff sessions were planned to be in-person, but the COVID-19 pandemic prompted a change to virtual participation beginning with cohort 2.

Kickoff day 1 consists predominantly of didactic presentations by the study investigators. First, participants are first given details about the study’s aims and procedures. Next, they receive an introduction to the coaching approaches that will be used throughout the regular dose intervention, the common rhetorical patterns of grant proposals, the value of oral processing and of engaging in iterative feedback cycles with a peer group during the writing process, and the strategic application of document design principles to increase clarity and reader comprehension. Day 1 concludes with each coaching group meeting in breakout rooms for introductions.

Coaching begins on kickoff day 2. After taking time to develop their meeting schedule for the next 5 months, each coaching group meets for consecutive 50-min segments. Each segment is dedicated to giving/receiving oral feedback on one participant’s draft of their specific aims page (and their biosketch, if time permits). If a participant is working on a resubmission, their NIH summary statement is the starting point of this discussion rather than the specific aims page. Participants email these documents to their coach and other group members 5 to 7 days before the kickoff. The kickoff concludes with a brief question and answer period with participants, coaches, and investigators. After participants disperse, coaches meet for 30 min with investigators to debrief on their initial coaching experiences.

##### Virtual coaching group meetings

**Fig. 2:**
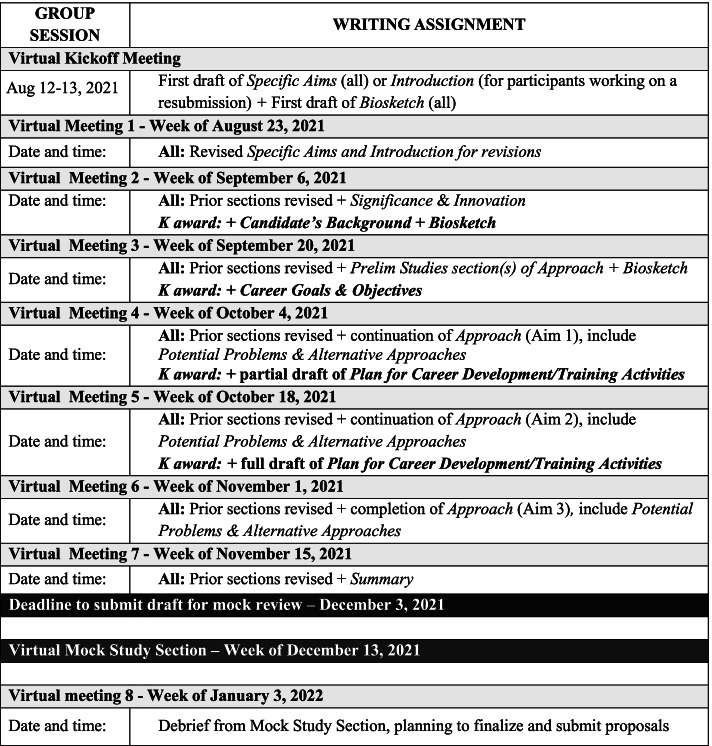
Example schedule for the regular dose coaching intervention. Each session is scheduled for 2 h, allowing for ~ 25 min of discussion per draft (assuming 5 participants per coaching group). Writing assignments can be adapted to adjust to participants’ unique needs, progress, and submission dates. However, all are expected to make progress and set a writing goal for each session. Participants submit their drafts to the coach and other group members a day or two before each meeting. As drafts become more complete, oral discussion focuses on the newest material

Following the kickoff, each group participates in eight, 2-h coaching sessions held approximately every 2 weeks by online video conference (e.g., Zoom). A sample schedule, including recommended writing assignments, is shown in Fig. 2. Prior to each meeting, participants share a draft of one or more sections of their proposal for review and oral critique by the coach and other group members during the virtual session. It is an explicit expectation that participants provide feedback on at least one of their peer’s drafts at every coaching session. Coaches may interact as needed with individual participants between meetings.

The coaching approaches applied during the regular dose intervention have been described in published reports on the NRMN phase 1 coaching models [7, 8]. Additionally, we provide excerpts from the study’s coaching manual as Additional File 2, which outline the core coaching practices and group facilitation techniques that coaches are expected to follow. The group coaching intervention is rooted in the premise that grant writing is a complex but teachable skill -- one that is best acquired through repetitive cycles of practice over a sustained period of active project development. Improvement during the proposal writing process is driven by input from highly skilled practitioners (grant writing coaches) and from additional readers (coaching group members, a content-aligned scientific advisor). Coaches who deliver the intervention do not need to be an expert in each participant’s research topic or approaches; however, all coaches possess deep knowledge of how reviewers evaluate a grant application and the implicit writing norms reviewers expect to be followed (e.g., limiting field-specific jargon; adhering to common rhetorical patterns, particularly on the specific aims page; including details that support the project’s feasibility and scientific rigor; crafting compelling statements about the expected impact of the research). The intervention promotes vicarious learning by situating writing-focused coaching within small peer groups. Participants quickly come to realize that they have a lot to contribute to improving the writing and the science of their peers. Participants can emulate strong writing examples from other group members, as well as avoid missteps and discuss challenges that arise during proposal development. Working in groups also fosters accountability and social support during the writing process.

##### Mock study section

After the completion of 7 virtual coaching sessions, each group conducts their own virtual mock study section. This is followed by a final, eighth coaching session, in which groups debrief from the mock study section and conclude the regular dose period. The majority of participants are expected to have developed a reasonably complete draft of their proposal (core scientific sections and biosketch, plus career development sections for K proposals) by the time their group’s mock study section takes place. If a participant’s progress is hindered such that their proposal is not well developed, then they participate in the session as observers.

Near the midpoint of the regular dose period, participants are prompted to consult with their scientific advisors to identify one person from within their professional networks who can provide an NIH-style written and oral review of their draft proposal. This external reviewer is expected to be familiar enough with the proposal’s scientific content to provide a realistic and rigorous review, but not be directly involved in the proposal’s development (e.g., as a K application mentor or a co-investigator). Reviewers participate in the group’s mock study section (a 30-min window) and in one optional follow-up phone call (if desired and initiated by the participant). The study provides reviewers with $200 for their service.

Procedures for the mock review session are outlined in Additional File 3. Reviewers receive their assigned proposal 7–10 days before the scheduled mock study session. They are instructed to critically read and score the draft proposal (for overall impact and individual criteria, using the NIH 1–9 scale), then prepare bulleted written comments (strengths, weaknesses) using a modified version of the relevant NIH review template. The coach facilitates the session, following a provided agenda. Each proposal is allocated 30 min of discussion. In the first 15 min, the external reviewer orally delivers a critique in the manner of a primary reviewer at an NIH study section meeting, while the participants and coach listen without comment. This is followed by 15 min of informal dialogue, during which the participant can ask clarifying questions about the review and the reviewer can offer suggestions for how to address the criticisms. The coach and other participants are also welcome to offer comments.

#### Extended dose coaching protocol

In the extended dose period, the group coaching process ends and is replaced by one-on-one support from the coach. The study supports a maximum of 10 h of individualized coaching per participant during the 18-month extended dose period. Additionally, $200 is provided for extended dose participants to receive one written mock review of their proposal, either before its initial submission or before its resubmission.

Although the frequency and type of extended dose coaching that takes place is largely participant driven, coaches and participants are prompted to connect at a few specific time points. At the start of the intervention, coaches contact each participant to ascertain proposal status and, for participants who have not yet submitted, develop a coaching plan to support completion of the grant application. Upon receipt of a summary statement from the NIH, extended dose participants are encouraged to meet with their coach to discuss the reviews and establish a revision plan. One study investigator is assigned to attend this meeting, providing another source of feedback at this critical time point.

### Engagement of scientific advisors in unstructured and structured arms

Across all study arms, scientific advisors are expected to work one-on-one with their participants during the 5-month regular dose period to provide scientific feedback on the grant proposal being developed. It is up to the discretion of each dyad to coordinate their interactions (how often to meet, type of feedback to provide, etc.).

In the unstructured engagement arms, the scientific advisor has no direct interaction with the coach or the coaching group. However, the coach and participant can discuss the feedback that the scientific advisor provides, and the coach can suggest specific issues or questions that a participant might bring to the advisor for input. In the structured engagement arms, scientific advisors are prompted to directly engage with the coaching group process at several time points. First, shortly after the regular dose kickoff session, advisors are asked to provide a brief written review of the participant’s specific aims page, which is then shared with the participant and the coach. Second, advisors are encouraged to actively participate in at least one of the eight coaching group meetings. Third, midway through the regular dose period, advisors are asked to take part in one phone call or virtual meeting with the participant and coach to check in on writing progress and discuss proposal-specific issues. Fourth, the advisor is invited to attend the group’s mock study section.

The continued involvement of scientific advisors during the extended dose is optional; each dyad decides whether and how to continue interacting with one another. If in the unstructured engagement arm during the regular dose, scientific advisors are to refrain from having any direct interaction with the coach during the extended dose.

### Coach selection and training for intervention delivery

The engagement of qualified coaches to deliver the interventions is a fundamental component of this study. For cohorts 1–3, we invited people who had served as successful grant writing coaches in at least one of the NRMN phase 1 coaching programs. These individuals are accomplished grant writers themselves (as judged by their past NIH funding and/or service on NIH review panels), are highly knowledgeable about best practices in grant writing, and have experience mentoring others in grant proposal development. Beginning with cohort 3, we had a need to re-use study coaches (those whose work with previous cohorts had concluded) or to recruit new coaches. To maximize available expertise while improving generalizability of our findings, we elected to do both. Study investigators invited people in their professional networks who had the required qualifications and agreed to be trained as coaches for the study. All coaches agree to be randomized to any of the four arms and receive a modest stipend for their participation.

We created a coaching manual that describes the study’s expectations, procedures, coaching methods, and group facilitation guidelines. Coaches participate in a 3-h orientation and training session to review the manual’s content and address any questions. The session is led by a subset of the study investigators before the start of the regular dose kickoff event. This training provides an opportunity for coaches to share their prior coaching and mentoring experiences with one another, while reinforcing the need for consistency in administering the study’s core coaching approaches. To confirm adherence to the protocol, one co-investigator attends a portion of each coaching group’s first session at the kickoff event and one additional coaching session about midway through the regular dose. If concerns with protocol deviations are identified, the coach is given feedback to redirect their efforts.

### Participant recruitment and eligibility criteria

Study recruitment for the first of our six planned cohorts began in October, 2019 and is ongoing. Information about the study and a link to its online application are housed on an NRMN-sponsored webpage. For each cohort, announcements about the study are distributed by email to members of the NRMN community and to other individuals or biomedical professional organizations with access to potential applicants.

Potential participants complete the online application form and upload three supporting documents: their NIH biosketch, a signed statement from their selected scientific advisor indicating agreement to participate and abide by the study’s protocol, and their scientific advisor’s NIH biosketch. Study investigators evaluate each written application to assess the applicant’s eligibility for the study, as defined by the following criteria:

- Early to mid-career faculty member developing a new or revised K-, R- or SC-series NIH proposal (or similar national-level proposal on a biomedical research topic); or a postdoctoral fellow developing a K99/R00 NIH proposal or different K-series NIH proposal designed to promote the transition to a faculty position.
- No prior R01 funding as a principal investigator.
- U.S. citizen or permanent resident.
- Proficiency to write a grant proposal in good scientific English (self-defined)
- Scientific ideas and preliminary data for the proposed research are sufficiently developed so that proposal writing can realistically begin at the start of the coaching intervention.
- Sufficient record of prior research training and publications to deliver a convincing argument for readiness to lead the proposed research.
- Intention to submit the proposal within 6 months after completion of the regular coaching dose.
- Identifies an appropriate Scientific Advisor who commits to providing critical feedback on the proposal’s scientific content during its development and engaging with the coaching process per randomization protocol.
- At an institution with sufficient resources (scientific, administrative) to support the proposed research, or has a strong collaboration with one or more scientists at such an institution.
- Has sufficient time to commit to full participation in all sessions, activities, and assignments for the study.

Since this is a pragmatic trial, we do not restrict participants from engaging in concomitant mentoring or other professional development activities during the trial. The one exception to this is that we exclude individuals who are actively engaged in a similar grant writing coaching intervention funded by the same U01 mechanism.

### Assignment to coaching groups

Eligible applicants are sorted by their project’s general research area and methods as specified in their application (quantitative versus qualitative; basic science, social and behavioral science, clinical science, data science, epidemiology, etc.), then assigned a random number. Applicants are tentatively assigned to coaching groups on this basis, with attention to ensuring representativeness by race/ethnicity and gender. When there are more eligible applicants than can be accommodated for a particular cohort, applicants are selected based on their random number ranking.

### Informed consent and enrollment

After applicants are selected and matched to a coaching group, they are contacted to provide informed consent and commit to the study’s expectations that all research ideas, materials, and discussions from the coaching intervention are kept confidential. Additionally, their scientific advisors are asked to confirm agreement to abide by their coaching group’s randomization. Participants are considered fully enrolled when all of these steps are completed.

### Coaching group randomization process

Each cohort comprises 8 to 20 coaching groups, in multiples of four. Within a cohort, entire coaching groups are randomized to one of the 4 study arms by using computer generated random numbers to assign equal numbers of groups to each arm. To mitigate potential statistical bias in this unblinded study, randomization assignment to the unstructured or structured arm is concealed until the kickoff, thus minimizing withdrawals related to the intervention group into which any individual was placed. Similarly, the randomization of groups to the regular dose or extended dose intervention is revealed after the end of the regular dose and after the majority of participants and coaches complete their post-coaching follow-up surveys and interviews (approximately 8–10 weeks after the regular dose ends).

### Participant withdrawal

Participants may choose to withdraw from the study for any reason at any time. Participants who fail to attend the required regular dose kickoff session are administratively withdrawn. After that time point, discontinued engagement or low levels of engagement with the coaching interventions are not a reason for withdrawal. When a participant indicates a desire to leave the coaching group, a co-investigator contacts them to request a brief interview. A scripted conversation guide is used to identify reasons for leaving and to determine whether study staff can continue to contact them in order to collect data on their future grant submissions and career development. A summary of this conversation is logged. Participants who chose to leave the study completely and not allow further data collection are withdrawn.

### Measures and data collection

Table 1 provides a summary of the study’s measures and data collection time points, from pre-intervention baseline assessments (time 0) through 24-month follow up. Data are collected and managed using REDCap (Research Electronic Data Capture) tools hosted at the University of Utah. REDCap is a secure, web-based data capture platform for research studies. It includes audit trails for tracking data manipulation as well as export procedures to common statistical software [26].

**Table 1:** Study Assessments and Data Acquisition Schedule

Timepoint (months):	Baseline/ Pre-Kickoff	Regular Dose Coaching Phase					Post	Extended Dose Coaching Phase and Follow up			
	0	1	2	3	4	5	6	7–11	12	18	24
**Participant Demographic and Background Variables (Survey)**											
Race, ethnicity, gender identity, disabilities, education	x										
Research training, scientific discipline, primary research area and methods	x										
Publications, previous grant writing experience	x										
**Participant Institutional Environment, Position Type (Surveys)**											
Access to mentoring and institutional research resources	x										
Institution, department, position, rank (faculty), appointment type (tenure, other)	x						x		x	x	x
Research/teaching/clinical-focused position; effort distribution across work roles	x						x		x	x	x
**Participant Outcome Variables (Surveys)**											
Primary: Funding of proposal(s) developed during coaching interventions							x		x	x	x
Secondary: Submission, scoring, resubmission of developed proposals							x		x	x	x
**Participant Other Assessments (Surveys)**											
Grant writing self-efficacy (19-CRAI)	x						x			x	x
Intention to pursue a biomedical research career (postdoctoral fellows only)	x						x		x	x	x
Self-efficacy to advance in career; scholarly activities to support advancement	x						x		x	x	x
Submission/funding of other proposals developed since participating in the study							x		x	x	x
Impact of COVID-19 pandemic on work life and grant writing (open-ended)^a^							x		x	x	x
**Participant Qualitative Assessments (Key Areas Addressed in Interviews)**											
Perceived value of coach and group meetings							x				x
Impact of group coaching group on grant writing process							x				x
Perceived value of peer feedback and mock review session							x				x
**Participant Feedback on Intervention (Surveys)**											
Perceived quality/value of coaching process, proposal feedback, other intervention components (individual items differ for the 6-month and 24 month surveys)							x				x
Satisfaction with scientific advisor: interaction frequency, feedback quality							x				x
**Process Measures (Coach, Participant, & Advisor Surveys; Coach Logs)**											
Participant attendance at group coaching sessions, completion of assignments, participants progress and barriers, number and type of coaching interactions outside of the group sessions		x	x	x	x	x					
Structured arms: Engagement of scientific advisors with coaching intervention		x	x	x	x		x				
Submission of proposal draft for group mock study section						x					
Frequency of scientific advisor interactions							x		x	x	x
Extended dose: Number and type of one-on-one coaching interactions, meetings to review summary sheets, and engagement of mock reviewers								x	x	x	
**Scientific Advisors: Demographics & Background, Feedback (Surveys)**											
Demographics, Institution and Position, Experience in Research and Mentoring	x										
Nature of relationship with participant (e.g., past/current mentor, colleague)	x										
Structured arm: Perceived value of direct engagement with coaching intervention							x				
Perception of the participant’s responsiveness to feedback							x				x
Self-assessment of their advising’s value to the proposal’s development							x				x
Expectation to continue in a professional relationship with the participant							x				x
**Coach Demographics & Background, Feedback (Surveys)**											
Demographics, Institution and Position, Experience in Research and Mentoring	x										
Perceptions of: their performance as a coach, quality of scientific advisor and peer feedback, value of other intervention components (e.g., mock review)							x				
**Coach Qualitative Assessments (Key Areas Addressed in Interviews)**											
Perceptions of group dynamics, peer feedback, and participant progress							x				
Perceived value of scientific advisor participation and mock reviews							x				
Perceptions of their contributions as coaches and the intervention’s impact on their mentoring practices							x				
Perceived value of the intervention to participants’ development							x				

^a^Assessed beginning with study cohort 2

#### Outcome measures

The primary outcome variable is participants’ success in acquiring external research grants, predominantly from the NIH, at 24 months after the initiation of regular dose coaching. Secondary outcomes include proposal submission rate, whether the proposal was discussed by the study section, proposal score after review, and resubmission rate. Proposal submission and review outcomes include both the proposal worked on during the regular dose coaching period as well as any other proposal submitted during the 2-year study timeframe. The coaching group is designed to teach the principles and skills of grant writing, which should transfer to future applications as well. Participants are late-stage post-doctoral fellows and early-career faculty who need to achieve grant funding success; pragmatically, they are involved in not only the focal proposal of this study but other proposals as a principal investigator or co-investigator, all of which may measure the effect of our interventions.

Additional outcomes that we are assessing include changes in participants’ perceived grant writing self-efficacy using the previously validated 19-question CRAI instrument [9], as well as three NRMN Common Measures addressing career intentions and self-efficacy to advance in one’s career. Lastly, open-ended questions about the impact of COVID-19 are included.

#### Structure and process measures

The fundamental grant writing coaching intervention under study is a complex, individually-tailored educational intervention administered by highly competent and experienced coaches. We described above the knowledge and experience that coaches must have in order to be selected to administer the study interventions, as well as the training we provide. Coaches are allowed flexibility in how they work with participants in their coaching groups. However, core components of the intervention’s structure and processes, including those that differentiate the study arms, are carefully described in the coaching manual, reiterated during the coach orientation and training session, presented to both coaches and participants during the regular dose kickoff session, and outlined in shared checklists for coaches, participants, and scientific advisors.

At the start of each cohort, coaches sign an agreement to abide by the randomization of their coaching group. Similarly, we document participants’ informed consent and confidentiality agreements, and scientific advisors’ signed agreement to abide by their participant’s randomization, as a criterion of dyad enrollment. Participants and coaches attend the regular dose kickoff session together, where they hear the same introduction to the study’s design and coaching interventions. Participants, scientific advisors, and coaches each receive arm-specific checklists summarizing our expectations for their activities during the regular dose period. These checklists are included as Additional File 4.

After each regular dose group coaching session (including the kickoff), coaches record attendance, document completion of assignments, and provide a brief assessment of each participant’s progress and barriers. Coaches log any substantive interactions with individual participants that take place outside of the scheduled group coaching sessions. In the structured engagement arms, coaches also record whether scientific advisors engaged as expected per the protocol. Co-investigators visit selected coaching sessions as observers to ensure that study processes are occurring as intended. Participants summarize their experiences with coaches, scientific advisors, and their peers during specific follow-up and feedback assessments. Scientific advisors report on their activities in brief surveys.

The extended dose phase of this study tests whether individualized coaching support after the end of the 5-month regular dose group coaching intervention improves participants’ success in grant funding over the 2 years following the kickoff. Brief manuals are provided to both coaches and participants (but not scientific advisors, as their participation is now optional). At the start of the extended dose period, coaches in these arms reach out to their group members and document the current status of their proposals (still in development, submitted, no longer pursuing). For participants who are still writing, the coach records the anticipated submission date and briefly outlines the plans they develop for working together to support that submission. Coaches log all of their coaching activities over the full 18 months. If a meeting takes place to discuss a submitted application’s reviews, the coach documents the outcome (discussed/not discussed, scores) and major critiques, then summarizes the recommendations and plans that are discussed for potential revision and resubmission. At the end of twenty-four months, participants and scientific advisors indicate in surveys how often they interacted since the end of the regular dose for the purpose of giving/receiving feedback on the proposal’s scientific content and the value of those interactions. They also indicate whether they will stay in contact as colleagues, which represents one indicator of the participant’s inclusion in a community of practice.

#### Quantitative data sources

Quantitative data for participants are collected from several sources. Demographic and background variables are extracted from participants’ applications to join the study and written baseline (pre-kickoff) surveys. Additional surveys are administered at the end of the regular dose (post), and at 12-, 18-, and 24-month follow up. These capture outcomes data (information about proposal submission, review, and funding decisions) as well as participant feedback on the coaching interventions.

Scientific advisors complete three brief written surveys. The first is a baseline survey that assesses demographics and background information, including the nature of their professional relationship with the participant (e.g., past or current mentor, colleague). The second and third surveys are completed at the end of the regular dose coaching sessions and at 24-month follow up, respectively. These instruments capture the frequency and type of interactions that advisors had with participants. They also document advisors’ self-assessment of the value they provided to participants during the proposal development process and whether they expect to continue in a professional relationship with the participant.

Coaches complete a written baseline survey that captures demographic variables and other descriptive background information. Coaches also indicate the types of research approaches and biomedical disciplines in which they feel they can effectively coach other grant writers. This information is used to match coaches to specific coaching groups. At the end of the regular dose, coaches complete a survey to obtain data on a variety of topics, including how the interventions were implemented and the degree to which each participant engaged in the groups.

#### Qualitative data sources

##### Participant interviews

Participants are interviewed at the end of the regular dose intervention. The post-regular dose interview guides are designed to draw out information revealing participants’ overall experiences with their coaching groups and factors impacting the development and submission of their proposals. The interview guides are the same for all four arms of the study, with the exception of specific questions related to scientific advisors’ structured engagement with the coaching process (for those randomized into a structured engagement arm). Interviews are semi-structured so as not to constrain the type of information that individuals feel are most important. The guide questions ensure that all of the same topics are opened up for each individual, but interviewers also probe for additional information and allow for exploration of emerging issues for each individual.

A key aim of the interview questions is to draw out and understand interactions among individuals, their coaches, and their coaching groups. The guide includes perceptions of the approaches of coaches, the dynamics of the coaching group, the benefits and drawbacks of working with the group, positive influences toward achieving their desired outcomes, and factors that may have inhibited their success. We anticipate a variety of small factors could impede a participant’s writing progress and proposal submission, but that in most cases a few contextual factors will drive the outcomes. Such factors may include lack of time to complete the proposal or insufficient preliminary data to enable a strong application. For any individual, the dominant factors could fall into any one or more of the domains of individual factors, group factors, or institutional factors.

Participants take part in a second interview near the end of their cohort’s two-year cycle. The 24-month interview guides are the same for all four arms of the study, with the exception of specific questions related to participants’ extended dose interactions with coaches (for those randomized to an extended dose arm). Because participants’ experiences and outcomes will not be known at the start of each interview, the guide is semi-structured with branching options to capture the most salient information for each participant. The primary goal of the interview is to elicit key information about what factors promoted or inhibited participants’ efforts to obtain research funding, and the role that the initial group coaching and/or extended dose individualized coaching played. Additionally, the interview is designed to reveal any significant changes in the person’s personal or professional situation since study enrollment.

We developed the interview guides by adapting a protocol used during the later stages of NRMN phase 1 (spring of 2018) to interview participants who were included in prior NRMN phase 1 coaching models but who had not yet obtained funding. This interview approach worked very well at that time and allowed us to identify many of the themes and factors that are being explored in the present study. Both interviews are expected to average 30 min in length. Careful attention is being paid to calibration among interviewers to ensure that all are collecting similar information using the guide. Interviews are audio recorded and immediately transferred through secure file transfer protocols to the University of Utah network file servers. They will be professionally transcribed, and the transcripts will serve as the basis of analysis.

##### Coach interviews

Coaches are interviewed at the end of the regular dose to capture their experiences and perceptions of both the coaching group process and the activities of their individual groups. Coaches who participate in more than one cohort are interviewed after each cohort. These semi-structured interviews are based on a guide that ensures the same topics are covered with each coach while providing sufficient latitude to capture different coaching group dynamics and activities. The interviews complement data obtained through the survey that coaches also complete at the end of the regular dose. In particular, the interviews are designed to elicit deeper insights into both individuals and group dynamics that can inform success or lack thereof for proposal completion, submission, funding outcomes, and subsequent resubmissions. The interviews also elicit information on the coach’s perceptions of the involvement of scientific advisors.

### Data analyses

Aim 1 of this study is to determine the effectiveness of a group coaching intervention when varying two factors: coaching dose (regular vs. extended) and mode of engaging participants’ scientific advisors with the coaching group process (unstructured vs. structured). The primary quantitative outcome is grant acquisition. Proposal submission is a secondary outcome. We hypothesize (non-null) that at 24 months, participants who receive the extended dose of grant writing coaching (Arms 3 & 4) will have significantly higher rates of funding than those who received the regular dose (Arms 1 & 2). Further, we hypothesize (non-null) that participants whose scientific advisors had structured engagement with the coaching process (Arms 2 & 4) will have significantly higher rates of funding than those who did not (Arms 1 & 3). Data on contextual factors are being collected from participants, scientific advisors, and coaches for descriptive purposes. This information will be used in auxiliary analyses when possible.

Aim 2 of this study is to conduct a qualitative evaluation of the success of the interventions based on content analysis of interviews of participants and coaches, to provide a deeper understanding of why, how, for whom, and in what contexts the study interventions are effective. The primary qualitative analysis approach is to code for emergent themes that arise in the interview transcripts. Our qualitative analyses will rely on an inductive, ‘constant comparison’ process [27]): we will not be testing any specific hypotheses and will avoid preconceived ideas of what we will find. Initially, several members of the research team with qualitative research expertise will independently read and annotate interview transcripts to identify and categorize key themes and patterns. We will construct a coding architecture gradually, using a ‘constant comparative’ process; study team members will identify preliminary themes in the transcripts and compare those themes across transcripts to identify patterns. Other key personnel will engage with the data analysis by reading and interpreting summaries of findings, including quotations upon which themes are based to ensure accuracy of coding and interpretation. Qualitative analysis will identify repeated themes as well as others occurring at lower frequencies but expressed by the interviewees as being critical to their experience. Our goal with the qualitative analysis is to reveal the complexity across the sample, by identifying emerging patterns and attending to individual experiences.

#### Quantitative analyses for aim 1

Aim 1 will provide a strong, statistically meaningful comparison of the 4 different study arms. A 2-sided test of equality of the intention-to-treat effectiveness of the extended-dose versus regular-dose coaching will be performed within a multi-level model for a dichotomous outcome [28]. Trainees are nested within coaching groups within cohorts and intervention arms. Intervention arms are crossed with cohorts. We will not analyze coach effects, as we anticipate that these will be negligible. We will perform intention-to-treat analysis by modern causal methods such as propensity-weighted analysis to produce an ‘average treatment effect’ [29, 30] if possible, or by traditional methods if necessary. An analysis will be informed by a directed acyclic graph [31] to identify the variables related to dropout. All tests will be 2-sided, at the 5% significance level. Similar analyses will be performed to evaluate the effectiveness of structured versus unstructured engagement of participants’ scientific advisors. We do not propose to test the statistical interaction between coaching dose and the scientific advisor’s mode of engagement as we expect them to be additive.

Ancillary and exploratory analyses will utilize multi-level models. As an ancillary analysis, we will calculate confidence intervals for grant success rates in each intervention arm, to compare to national rates for first-time grant writers and other benchmarks. If sample sizes and statistical assumptions permit, we will also investigate the success rates of URM participants versus non-URMs; participants from minority serving institutions versus other institutions; and participants from different disciplines, on an exploratory basis. We will also test the effects of the study interventions on NIH overall impact scores as an ordinal outcome. Where intermediate mechanisms such as grant writing self-efficacy can be operationalized, we will plot time trends, and report associations with study interventions and with outcomes, with further analysis by multilevel structural equations models if possible.

#### Adequacy of sample size for quantitative analysis

For our first hypothesis, we made the following assumptions: a funding success rate of 15% in the regular dose arms (conservatively based on the historical success of participants in the phase 1 NRMN grant writing coaching models); an intraclass correlation within coaching groups of 0.10 (a size commonly found in the medical literature); and an 80% power at the 5% significance level. We used the simple design effect of Donner and Klar [32] and PASS 2011 software as an approximation to the sample size calculation for this multilevel analysis. With a total of 72 coaching groups of 4 or 5 participants/group (36 coaching groups per level of an intervention arm, in clusters of size 4 to 5), we have 80% power to detect an approximately 30% success rate in the extended-dose arms versus a 15% success rate in the regular dose arms, at the 5% significance level. Power for our second hypothesis is anticipated to be similar, assuming that the unstructured engagement arm will have a funding success rate of 15%.

#### Qualitative analyses for aim 2

Aim 2 will focus on understanding the mechanisms behind any detected differences between the 4 arms, as well as within-arm differences. A qualitative analysis of semi-structured trainee and coach interviews will provide a trustable “thick description” [33] of why, how, for whom, and in what contexts interventions worked.

For participant interviews, the qualitative approach for this study is to draw out consistent information from participants, to allow for identification of patterns, while allowing flexibility to identify unique situations or factors for each person. We will study three broad domains: 1) factors associated with an individual, both current and past situations; 2) factors associated with their interaction with the intervention, such as the effectiveness of their coach and group members; 3) their institutional setting, that is, the context within which they operate. Within these three domains we will examine factors aligned with submission (or not) of an initial proposal; resubmission if not funded; and successful funding. In essence, the factors can be thought of within a 3 X 3 matrix but without necessarily information related to each person in each matrix element.

Coach interviews will be analyzed from several perspectives through the ‘eyes’ of the coaches. First, are there discernable differences in scientific advisor participation and contributions between structured and unstructured participation? Second, what are the ranges and/or commonalities of dynamics across the many different coaching groups in the study? What seemed to make groups effective and highly functional versus less effective? Are the coaches able to identify critical factors leading to success in proposal submission and funding at the participant or institutional level? At the individual participant level, are coaches able to pinpoint what promotes or impedes success? When coaches reflect on the overall design of the coaching intervention, are there elements that they feel are critically important for success? By contrast, are there things they would change if adapting the coaching group design into practice (e.g. is the high expectation for keeping up with a new section to review every 2 weeks promoting progress or too demanding ca using some to ‘withdraw’ either formally or informally)?

## Discussion

Despite growth in training efforts (e.g., by universities, grant-funded organizations, for-profit individuals and groups) to enhance the grant writing success of early-career biomedical investigators, few systematic studies have rigorously tested the effectiveness of such endeavors or evaluated the components or factors that contribute to program effectiveness. Our group-randomized trial is testing well-defined variations of an enhanced, grant writing group coaching intervention to identify features that influence its impact. These features include the modifiable contributions of coaching dose/duration and the level of integration of scientific content advisors (typically a local content-aligned mentor or colleague) into the group coaching program. The design of our refined intervention -- and the selection of which intervention features to test -- was informed by our previous work and a desire to increase the generalizability and future translation of our findings. The intervention we are testing is highly replicable and scalable within and between institutions. Study findings will produce practical information to guide implementation of the tested intervention by others. This information is critical, as our group coaching approach is substantially different from brief workshop-based trainings and from the typical one-on-one engagement of a mentor/mentee dyad during a grant proposal’s development.

Importantly, our study design allows us to identify the more nuanced individual-, coaching group-, and institutional-level factors that contribute to proposal submission and grant acquisition among people who engage in this type of intervention. For example, the qualitative data we collect comparing the regular and extended dose will provide insight into the reasons why participants fail to complete and submit their grant applications, even when provided with substantive coaching during the writing process. Identifying those barriers could influence the way that fellows/faculty and their institutional mentors approach the grant writing process. Further, we expect that study interviews will generate a variety of perspectives on how to further optimize the coaching approaches. Given the already high demands of the participants that this intervention targets and the coaches who deliver it, maximizing the efficiency of intervention delivery is a high-value outcome.

An important feature of our study is intentional oversampling of participants from URM backgrounds. Studies have shown that URM scientists are disproportionately less likely to be awarded R01 research grants by the NIH. Previous work by our team showed that URM investigators who engaged in grant writing coaching groups have similar rates of grant submission and funding compared to non-URM investigators [8]. Having URM participants in sufficient proportion will provide additional insights and validation of data about factors associated with intervention success.

One of the operational issues we face in conducting this study is accurately assessing whether an individual will be sufficiently “ready to write” at the start of the regular dose coaching period. This is important, as the intense and consistent writing required by the intervention is not realistic for someone whose ideas are just forming or who lacks the preliminary data needed to define the core direction their research will take. Our screening on this eligibility criterion has inherent limitations, because it relies on participants’ self-reporting about the depth of their project ideas and availability of sufficient preliminary data. When eligibility on this measure is uncertain, multiple study investigators meet to discuss the application until a consensus is reached. However, even with this screening, some individuals may enter the study at a very preliminary stage of a proposal’s development, which could affect their engagement and ability to benefit.

A second practical issue is that once enrolled in the study, participants may face challenges to fully engaging in the coaching intervention. Obvious obstacles are time demands and unexpected complications such as those precipitated by the COVID-19 pandemic. At the regular dose kickoff, participants are encouraged to remain engaged as they are able, with the understanding that grant writing skills can be enhanced even when progress is slower than hoped. During coach training, we emphasize the importance of cultivating a group coaching environment that is collegial and supportive, particularly when participants are struggling in some way.

A third issue is that data collection for each cohort ends at 24 months after initiation of the regular dose intervention. We selected this time point so that data collection could be completed by the end of the project’s funding period. However, we know the process of submission, review, and resubmission of an NIH proposal often exceeds that timeframe. Therefore, complete data on our primary outcome measure of proposal funding rates will not be available at the study’s conclusion. Additional time and resources will be needed to capture outcomes beyond this timeframe.

In conclusion, successful grant writing efforts provide biomedical investigators with the resources they need to build their research programs, train future researchers, and contribute to the health of the world population. Results of this study will provide a much needed empirical understanding of how organizations might feasibly enhance the grant writing activities of their faculty, fellows, and other research trainees, including those from URM backgrounds.

## Supplementary Information

**Additional file 1.** Agenda for Kickoff Session to Regular Dose Coaching Intervention.

**Additional file 2.** Excerpts from the Regular Dose Coaching Manual.

**Additional file 3.** Schedule and Procedures for the Mock Study Section.

**Additional file 4.** Arm-specific Checklists for Coaches, Participants, and Scientific Advisors.

**Additional file 5.** Study Assessments and Data Acquisition Schedule.

## Data Availability

Not applicable; this study protocol article does not contain any data.

## Abbreviations

NIH: National Institutes of Health
NRMN: National Research Mentoring Network
URM: Under-represented racial/ethnic minority
