# Supplementary material for: Variations of a group coaching intervention to support early-career biomedical researchers in Grant proposal development: a pragmatic, four-arm, group-randomized trial
Source: BMC Med Educ. 2022 Jan 10;22:28. doi: 10.1186/s12909-021-03093-w (PMC8744062; doi:10.1186/s12909-021-03093-w)
Supplement: Supplementary file 1 — Additional file 1. Agenda for Kickoff Session to Regular Dose Coaching Intervention. [file 12909_2021_3093_MOESM1_ESM.pdf]

Additional File 1

Agenda for Kickoff Session to Regular Dose Coaching Intervention

Day 1

Time	Topic
11:00 am - 11:30 am	**Coaches only:** Check in, question and answer with study investigators
11:30 am - 12:00 pm	**Participants only:** Technology check
12:00 pm	Welcome, Introduction to the study
12:30 pm	Introduction of coaches
12:45 pm	Introduction to the coaching intervention
1:10 pm	10-minute stretch break
1:20 pm	Introduction to writing for reviewers, rhetorical patterns, oral feedback
2:00 pm	Grant proposal design considerations
2:30 pm	Break into coaching groups for introductions
3:30 pm - 4:30 pm	Optional networking and socializing

Day 2

Time	Topic
8:45 am	Introduction to the day
8:55 am	Groups create schedule for virtual coaching sessions
9:10 am	Aims review and feedback, Participant #1
10:00 am	10-minute stretch break
10:10 am	Aims review and feedback, Participant #2
11:00 am	10-minute stretch break
11:10 am	Aims review and feedback, Participant #3
12:00 pm	Lunch break
12:30 pm	Aims review and feedback, Participant #4
1:20 pm	10-minute stretch break
1:30 pm	Aims review and feedback, Participant #5
2:20 pm	10-minute stretch break
2:30 pm	Closing remarks , question and answer with study investigators
2:45 pm - 3:15 pm	**Coaches only**: Debrief with study investigators
