# Supplementary material for: Variations of a group coaching intervention to support early-career biomedical researchers in Grant proposal development: a pragmatic, four-arm, group-randomized trial
Source: BMC Med Educ. 2022 Jan 10;22:28. doi: 10.1186/s12909-021-03093-w (PMC8744062; doi:10.1186/s12909-021-03093-w)
Supplement: Supplementary file 2 — Additional file 2. Excerpts from the Regular Dose Coaching Manual. [file 12909_2021_3093_MOESM2_ESM.pdf]

Additional File 2

Excerpts from the Regular Dose Coaching Manual

I. What are my roles as a coach in this study?

Your overarching role is to support participants' timely development of high quality grant applications by reviewing and providing feedback on their developing drafts in a small group setting over several months, and by sharing your advice and wisdom on topics related to becoming a successful grant writer. You will perform your coaching role in the context of a specific intervention that we are testing in this study.

II. What is the coaching intervention's intended purpose?

The coaching intervention is designed to do the following:

- Enhance participants' grant writing knowledge and skills by applying core tenets of effective teaching and learning (such as systematic and deliberative practice, rapid feedback from experts, vicarious learning by participating in practice cycles with others).
- Deconstruct the genre of grant writing and elucidate its common rhetorical patterns.
- Educate participants about reviewers' content expectations for different sections of a grant application.
- Expose participants to readers' detailed thought processes and reactions to drafted text.
- Encourage smart document design to improve clarity and comprehension.
- Normalize the challenges of grant writing and minimize social stresses.
- Provide structure and accountability to support writing progress.
- Facilitate pre-submission review of draft applications by others within and outside of participants' specific fields.
- Encourage timely submission and resubmission of proposals.

III. How do I conduct the regular dose virtual group coaching sessions?

A. Preparing for each coaching session:

1) Solicit updated proposal drafts from all group participants in advance of each session (we suggest 1-2 days prior).
2) Ask that participants always submit their full proposal-in-progress, but indicate the new areas of text that they would like to get the most feedback on.
3) Share the proposal drafts with your group via email, a shared folder, or other process that the group agrees on.
4) Assign one draft to each participant, with the expectation that they will be asked to provide some oral feedback on their assigned draft during the next coaching session.
5) Familiarize yourself with each draft. If you are unfamiliar with the specific mechanism and/or program announcement for which the proposal is being written, be sure to review that information.

B. Facilitating each coaching session:

Core coaching expectations:

1) Emphasize to participants that their proposal drafts and their interactions with you and other group members are confidential and privileged information. No one is to share written materials or discuss others' specific research ideas outside of the coaching sessions. Further, it is unethical to take the research ideas of other participants and apply them in their own research without explicit permission.
2) Maintain a positive, supportive, and respectful environment. This is essential for group cohesion, engagement, learning, and productivity.
3) Divide up the meeting to give equal time for discussion of participants' drafts.
4) Apply a mix of coaching strategies for analyzing the draft and offering feedback. These strategies might include the following:
   - *Engaging in "real time oral processing": Voicing issues and questions that come to mind as you read the draft (related to either the science or the*

writing) - e.g. "This is what my brain was thinking when I was reading this, this is what I found myself wondering, not understanding";

- *Clarifying who is likely to review the submitted proposal (e.g., composition of study section) so that the feedback you and group members provide is on target for likely reviewers, not readers who are too far outside the field;*
- *Articulating whether more or less detail might be needed in the text, especially in the Approach section (newer investigators often need guidance on what kind and how much detail reviewers expect to see for different kinds of study designs and research methods);*
- *Identifying problematic prose in specific sections or recurring writing-related issues in the document and offering suggestions for improvement;*
- *Giving an honest assessment of how well the draft meets the review criteria;*
- *Recommending specific issues or questions that participants should talk about with their Scientific Advisors.*

5) Provide a balance of positive comments (it is always helpful to start and end with these!) and more critical feedback.
6) Focus your critique on the newest sections of the draft before commenting on previously drafted material.
7) Focus on core content concerns and major issues before addressing minor, easily fixable problems.
8) Deliver your feedback <u>and</u> solicit feedback from other group members.
9) Encourage participants to carefully listen to and digest the feedback they are receiving in real time. As comments are shared, participants should ask questions to help them understand the problems or questions being raised by readers and the revisions being suggested. Because participants will have access to the full meeting recordings and transcripts, they should be more engaged in the discussion itself rather than on note taking during the meeting.
10) Encourage questions and informal discussion. The more engaged participants are in examining each other's proposals and asking questions in the moment, the more shared learning occurs. However, take care to balance this with an eye on the clock to make sure a person's dedicated review time is not taken over by tangential discussion.

11) Welcome and create opportunities to explore relevant grant topics such as identifying study sections, finding collaborators, composing a mentoring team for a K award, etc. Participants will likely broach topics themselves, but you are encouraged to proactively introduce them as appropriate windows arise.

12) Conclude each coaching session by reminding participants about the next writing assignment and encouraging them to engage with Scientific Advisors.

Additional coaching recommendations:

1) Regular or occasional written feedback on drafts is welcome, but not required of coaches or participants. We strongly discourage coaches or participants from using the valuable group coaching time to verbally walk through a comprehensive list of comments and line edits from any document editing they might have done. This type of detailed feedback is better shared via email.

2) Do not shy away from asking tough questions or voicing major concerns about the project being proposed (the science, the project's significance, scope of work) or its written presentation. Honest and constructive criticism is essential for participants to gain the most benefit from the coaching intervention.

3) Do not hesitate to raise questions and concerns (either scientific or writing related) for which you do not have an immediate solution. Pointing out potential problems has inherent value. Others in the group might have ideas for how to address the issue. In some cases the participant will need to explore solutions on their own or in consultation with their Scientific Advisor.

4) Be open to differences of opinion among the group in suggestions for revision. However, if a participant gives feedback to another group member that you know is incorrect or think is misguided, tactfully point this out and explain why.

5) Some participants struggle with receiving a lot of feedback at once or addressing conflicting comments. Remind them that they can talk with you or their Scientific Advisors to help them sort through the feedback and make a revision plan, and that they as the principal investigators have the final say in what their projects and proposals will look like.

6) Be generous and deliberate in highlighting examples of exciting scientific ideas, well-crafted prose, figures and tables, document design, etc. from participants' drafts. A tangible benefit of the group coaching model is that participants see each other's work in progress and can apply successful writing strategies used by others.
