# Supplementary material for: Variations of a group coaching intervention to support early-career biomedical researchers in Grant proposal development: a pragmatic, four-arm, group-randomized trial
Source: BMC Med Educ. 2022 Jan 10;22:28. doi: 10.1186/s12909-021-03093-w (PMC8744062; doi:10.1186/s12909-021-03093-w)
Supplement: Supplementary file 3 — Additional file 3. Schedule and Procedures for the Mock Study Section. [file 12909_2021_3093_MOESM3_ESM.docx]

Additional File 3

**Agenda and Procedures for the Mock Study Section**

| **Written Critique:** | Reviewer emails this to the participant and coach just before or just after the mock study section |
| --- | --- |

| **Time** | **Reviewer name** | **Reviewer email** | **Participant name** | **Participant email** | **Application Type, Working Title** |
| --- | --- | --- | --- | --- | --- |
| 9:00-9:30 |  |  |  |  |  |
| 9:30-10:00 |  |  |  |  |  |
| 10:00-10:30 |  |  |  |  |  |
| 10:30-11:00 |  |  |  |  |  |
| 11:00-11:30 |  |  |  |  |  |

**Procedures for each 30-minute review block:**

2 min: Coach welcomes and introduces reviewer, explains procedures.

15 min: Reviewer delivers NIH-style oral review and scores while coaching group listens.

10 min: Group discussion, facilitated by coach

- Focus of discussion is Q&A between participant and reviewer.
- **Participant** is encouraged to ask clarifying questions about the review, as well as solicit feedback on any application content or issues not mentioned in the review.
- **Reviewer** offers recommendations for how to address criticisms.
- **Coach and other participants** can contribute to discussion, offering comments on the proposal, asking questions about the critique or recommended revisions, etc.

3 min: Coach wrap up: Thank you, reminder that the reviewer has agreed to participate in one follow up phone call with the participant for a deeper discussion. This call is optional, participant will contact reviewer to initiate this if desired.
