# Supplementary material for: Variations of a group coaching intervention to support early-career biomedical researchers in Grant proposal development: a pragmatic, four-arm, group-randomized trial
Source: BMC Med Educ. 2022 Jan 10;22:28. doi: 10.1186/s12909-021-03093-w (PMC8744062; doi:10.1186/s12909-021-03093-w)
Supplement: Supplementary file 4 — Additional file 4. Arm-specific Checklists for Coaches, Participants, and Scientific Advisors. [file 12909_2021_3093_MOESM4_ESM.pdf]

Additional File 4

## Arm-Specific Checklists for Coaches, Participants, and Scientific Advisors

### Coach Checklist - Unstructured Arm

**At kickoff:**

- ☐ Work with your group to (a) define schedule for virtual coaching meetings and (b) confirm availability for mock study section date/time slots. Send completed schedule and mock study section availability forms to study staff before the end of the kickoff. Block these dates and times on your calendar.

---

**Throughout the coaching intervention:**

- ☐ *DO NOT have any direct contact with Scientific Advisors (email, phone calls, virtual coaching sessions, mock review, etc).*

---

**Before each coaching session:**

- ☐ Solicit updated drafts from group participants and assign each participant to review one draft.

**During each coaching session:**

- ☐ Encourage participants to share feedback from the coaching session with their Scientific Advisors (and vice versa), and to regularly consult with their Advisors.

**After each coaching session:**

- ☐ Within 24 hours, complete the "Progress and Barriers" form (link will be emailed to you by study staff) to record attendance and briefly report on participants' progress and barriers.

**Between coaching sessions:**

- ☐ Remind participants of upcoming sessions and assignments.
- ☐ Be responsive to participants' questions and engage as needed in additional coaching interactions to best support the progress of individual group members.
- ☐ Use the study spreadsheet to report any substantive coaching interactions that you have with participants outside of the virtual coaching meetings.

---

**Miscellaneous tasks:**

- ☐ Help participants find a suitable mock reviewer (as needed and able). Facilitate your group's Mock Study Section.
- ☐ Be responsive to requests from staff regarding additional study tasks, such as planning for the mock study section.
- ☐ Complete study assessments (surveys, interviews) as prompted by study staff.

## Participant Checklist - Unstructured Arm

**At kickoff:**

- ☐ Work with your coach and other group members to (a) define schedule for virtual coaching meetings and (b) confirm availability for mock study section date/time slots. Block these dates and times on your calendar.

---

**Before each coaching session:**

- ☐ Complete and submit your writing assignment by the group's agreed upon deadline.
- ☐ Read the one participant draft assigned to you by your coach and prepare to deliver some oral feedback on that draft.

**During each coaching session:**

- ☐ Actively listen to and ask questions about the feedback you are receiving from your coach and other group members.
- ☐ Offer feedback on the drafts of other participants.

**Between coaching sessions:**

- ☐ Carefully review and consider the feedback that you received during the last session.
- ☐ Share feedback with your Scientific Advisor and regularly seek their scientific input. It is up to you and your Advisor to decide how frequently you will meet as a dyad and how to structure your interactions.
- ☐ Complete revision/writing assignment for the next coaching session.
- ☐ Seek additional guidance from your coach and ask questions as needed to help you make progress on your application.

---

**For the Virtual Mock Study Section:**

- ☐ Work with your Scientific Advisor to identify another scientist in your discipline who is willing to review your draft grant application at the Mock Study Section.

---

**Miscellaneous tasks:**

- ☐ Complete study assessments (surveys, interviews) as prompted by study staff.

# Scientific Advisor Checklist - Unstructured Arm

**Throughout the 6-month coaching intervention:**

- ☐ Provide the participant with ongoing, one-on-one, scientific feedback on their developing grant proposal. It is up to you and the participant to decide on how frequently you will meet as a dyad and how to structure your interactions.
- ☐ Encourage the participant to share feedback from the coaching session with you (and vice versa); offer assistance with responding to this feedback as needed and as your time allows.
- ☐ *DO NOT have any direct contact with the grant writing coach or coaching group.*

---

**For the Virtual Mock Study Section:**

- ☐ Identify other scientists in your discipline who might be qualified and willing to review the participant's draft grant application.

---

**Miscellaneous tasks:**

- ☐ Complete study assessments (surveys) as prompted by study staff.

## Coach Checklist - Structured Arm

**At kickoff**:

- ☐ Work with your group to (a) define schedule for virtual coaching meetings and (b) confirm availability for mock study section date/time slots. Send completed schedule and mock study section availability forms to study staff before the end of the kickoff. Block these dates and times on your calendar.

**Between the kickoff and 1st virtual coaching session:**

- ☐ Remind participants to share the coaching schedule with their Scientific Advisors, to ask their Advisors to select 1-2 virtual coaching meetings to attend, and to share those selections with you.
- ☐ Remind participants to send you their revised Specific Aims pages and their Advisors' brief written review of that page by the agreed upon deadline.

**Approximately midway through the coaching schedule:**

- ☐ Schedule and conduct one 30-minute three-way conversation with each dyad (participant and Scientific Advisor) in your group to check in on progress and discuss proposal-specific issues.

---

**Before each coaching session:**

- ☐ Solicit updated drafts from group participants, assign each participant to review one draft
- ☐ Confirm which Scientific Advisors will be attending the upcoming session.

**During each coaching session:**

- ☐ When Advisors are present, engage them in the discussion.
- ☐ When Advisors are absent, ask participants to briefly report on any recent conversations they have had with their Advisors. If necessary, strategize with participants about how to more effectively engage their Advisors.
- ☐ Encourage participants to share feedback from the coaching session with their Scientific Advisors and to regularly consult with their Advisors.

**After each coaching session:**

- ☐ Within 24 hours, complete the "Progress and Barriers" form (link will be emailed to you by study staff) to record attendance and briefly report on participants' progress and barriers.

**Between coaching sessions:**

- ☐ Remind participants of upcoming sessions and assignments.
- ☐ Be responsive to participants' questions and engage as needed in additional coaching interactions to best support individual group members.
- ☐ Use the coaching log to report any substantive coaching interactions you have with participants (and others, such as Scientific Advisors) outside of the virtual coaching meetings.

---

**Miscellaneous tasks:**

- ☐ Help participants find a suitable mock reviewer (as needed and able). Facilitate your group's mock study section.
- ☐ Be responsive to requests from staff regarding additional study tasks, such as planning for the mock study section.
- ☐ Complete study assessments (surveys, interviews) as prompted by study staff.

## Participant Checklist - Structured Arm

**At kickoff**:

- ☐ Work with your coach and other group members to (a) define schedule for virtual coaching meetings and (b) confirm availability for mock study section date/time slots. Block these dates and times on your calendar.

**Between the kickoff and 1st virtual coaching session:**

- ☐ Apply feedback from the kickoff coaching session to revise your Specific Aims page.
- ☐ Share the revised Specific Aims with your Scientific Advisor and request a brief written review of these, using the template provided by the study.
- ☐ Submit your revised Specific Aims <u>and</u> your Advisor's written review to your coach by the agreed upon deadline.
- ☐ Share your group's coaching schedule with your Scientific Advisor and ask them to select at least one of the virtual coaching meetings to attend, preferably two. If only one is possible, prioritize a session when the Approach section of the grant application will be discussed (refer to schedule template).
- ☐ Tell your coach which meeting(s) your Advisor has selected to attend.

**Approximately midway through the coaching schedule:**

- ☐ Participate in a 30-minute three-way conversation with your coach and Scientific Advisor to check in on your progress and discuss proposal-specific issues. Your coach is responsible for scheduling this call.

---

**Before each coaching session:**

- ☐ Complete and submit your writing assignment by the group's agreed upon deadline.
- ☐ Alert your coach if your Scientific Advisor plans to attend the upcoming session.
- ☐ Read the one participant draft assigned to you by your coach and prepare to deliver some oral feedback on that draft.

**During each coaching session:**

- ☐ Briefly report on any recent conversations you have had with your Scientific Advisor.
- ☐ Actively listen to and ask questions about the feedback you are receiving from your coach and other group members.
- ☐ Offer feedback on the drafts of other participants.

**Between each coaching session:**

- ☐ Carefully review and consider the feedback that you received during the last session.
- ☐ Share feedback with your Scientific Advisor and regularly seek their scientific input. It is up to you and your Advisor to decide how frequently you will meet as a dyad and how to structure your interactions.
- ☐ Complete revision/writing assignment for the next coaching session.
- ☐ Seek additional guidance from your coach and ask questions as needed to help you make progress on your application.

---

**For the Virtual Mock Study Section:**

- ☐ Work with your Scientific Advisor to identify another scientist in your discipline who is willing to review your draft grant application at the Mock Study Section.

---

**Miscellaneous tasks:**

- ☐ Complete study assessments (surveys, interviews) as prompted by study staff.

## Scientific Advisor Checklist - Structured Arm

**Between the kickoff and 1st virtual coaching session:**

- ☐ Review the coaching group's virtual meeting schedule that was developed at the kickoff (to be provided by the participant). Select at least one of the virtual coaching meetings to attend, preferably two. If only one is possible, prioritize a session when the Approach section of the grant application is likely to be discussed (refer to schedule template). Share your selections with the participant and block these on your calendar.
- ☐ Read the participant's revised Specific Aims page (to be provided by the participant).
- ☐ Write a short (~half page) critique of this Specific Aims page (using the provided template); give your completed review to the participant by the agreed upon deadline. The participant will share your written review with the coach.

---

**Throughout the 6-month coaching intervention:**

- ☐ Provide the participant with ongoing, one-on-one, scientific feedback on their developing grant proposal. It is up to you and the participant to decide on how frequently you will meet as a dyad and how to structure your interactions.
- ☐ Encourage the participant to share feedback from the coaching session with you (and vice versa); offer assistance with responding to this feedback as needed and as your time allows.

---

**Before the virtual coaching session(s) that you selected to attend:**

- ☐ Read the participant's most up-to-date proposal draft and prepare to deliver some oral feedback on the draft during the coaching session.

**During the virtual coaching session(s) that you selected to attend:**

- ☐ Actively engage in the coaching group's facilitated discussion about your participant's draft.

---

**Approximately midway through the coaching schedule:**

- ☐ Participate in a 30-minute three-way conversation with the participant and coach to check in on progress and discuss proposal-specific issues. The coach is responsible for scheduling this call.

---

**For the Virtual Mock Study Section:**

- ☐ Identify other scientists in your discipline who might be qualified and willing to review the participant's draft grant application.
- ☐ Participate in the virtual mock study section by reviewing the participant's draft proposal in advance, calling in to the meeting during the participant's review window, listening to the reviews, and offering your own feedback.

---

**Miscellaneous tasks:**

- ☐ Complete study assessments (surveys) as prompted by study staff.
