# Supplementary material for: Variations of a group coaching intervention to support early-career biomedical researchers in Grant proposal development: a pragmatic, four-arm, group-randomized trial
Source: BMC Med Educ. 2022 Jan 10;22:28. doi: 10.1186/s12909-021-03093-w (PMC8744062; doi:10.1186/s12909-021-03093-w)
Supplement: Supplementary file 5 — Additional file 5. Study Assessments and Data Acquisition Schedule. [file 12909_2021_3093_MOESM5_ESM.pdf]

Table 1: Assessments and Data Acquisition Schedule

	Baseline/ Pre-Kickoff	Regular Dose Coaching Phase					Post	Extended Dose Coaching Phase and Follow up			
**Timepoint (months):**	**0**	**1**	**2**	**3**	**4**	**5**	**6**	**7-11**	**12**	**18**	**24**
**Participant Demographic and Background Variables (Survey)**											
Race, ethnicity, gender identity, disabilities, education	x										
Research training, scientific discipline, primary research area and methods	x										
Publications, previous grant writing experience	x										
**Participant Institutional Environment, Position Type (Surveys)**											
Access to mentoring and institutional research resources	x										
Institution, department, position, rank (faculty), appointment type (tenure, other)	x						x		x	x	x
Research/teaching/clinical-focused position; effort distribution across work roles	x						x		x	x	x
**Participant Outcome Variables (Surveys)**											
Primary: Funding of proposal(s) developed during coaching interventions							x		x	x	x
Secondary: Submission, scoring, resubmission of developed proposals							x		x	x	x
**Participant Other Assessments (Surveys)**											
Grant writing self-efficacy (19-CRAI)	x						x			x	x
Intention to pursue a biomedical research career (postdoctoral fellows only)	x						x		x	x	x
Self-efficacy to advance in career; scholarly activities to support advancement	x						x		x	x	x
Submission/funding of other proposals developed since participating in the study							x		x	x	x
Impact of COVID-19 pandemic on work life and grant writing (open-ended)*							x		x	x	x
**Participant Qualitative Assessments (Key Areas Addressed in Interviews)**											
Perceived value of coach and group meetings							x				x
Impact of group coaching group on grant writing process							x				x
Perceived value of peer feedback and mock review session							x				x
**Participant Feedback on Intervention (Surveys)**											
Perceived quality/value of coaching process, proposal feedback, other intervention components (individual items differ for the 6-month and 24month surveys)							x				x
Satisfaction with scientific advisor: interaction frequency, feedback quality							x				x

**Process Measures (Coach, Participant, & Advisor Surveys; Coach Logs)**											
Participant attendance at group coaching sessions, completion of assignments, participants progress and barriers, number and type of coaching interactions outside of the group sessions		x	x	x	x	x					
Structured arms: Engagement of scientific advisors with coaching intervention		x	x	x	x		x				
Submission of proposal draft for group mock study section						x					
Frequency of scientific advisor interactions							x		x	x	x
Extended dose: Number and type of one-on-one coaching interactions, meetings to review summary sheets, and engagement of mock reviewers								x	x	x	
**Scientific Advisors: Demographics & Background, Feedback (Surveys)**											
Demographics, Institution and Position, Experience in Research and Mentoring	x										
Nature of relationship with participant (e.g., past/current mentor, colleague)	x										
Structured arm: Perceived value of direct engagement with coaching intervention							x				
Perception of the participant's responsiveness to feedback							x				x
Self-assessment of their advising's value to the proposal's development							x				x
Expectation to continue in a professional relationship with the participant							x				x
**Coach Demographics & Background, Feedback (Surveys)**											
Demographics, Institution and Position, Experience in Research and Mentoring	x										
Perceptions of: their performance as a coach, quality of scientific advisor and peer feedback, value of other intervention components (e.g., mock review)							x				
**Coach Qualitative Assessments (Key Areas Addressed in Interviews)**											
Perceptions of group dynamics, peer feedback, and participant progress							x				
Perceived value of scientific advisor participation and mock reviews							x				
Perceptions of their contributions as coaches and the intervention's impact on their mentoring practices							x				
Perceived value of the intervention to participants' development							x				

***Assessed beginning with study cohort 2**
